# 272. Dissecting Host-Commensal Interactions During Early Life to Identify Mechanisms of Protection Against Autoimmune Diabetes

**DOI:** 10.1093/ofid/ofad500.344

**Published:** 2023-11-27

**Authors:** Logan Grimes, Julia Flores, J B Lubin, Jamal Green, Michael Silverman

**Affiliations:** Children's Hospital of Philadelphia, Philadelphia, Pennsylvania; Children's Hospital of Philadelphia, Philadelphia, Pennsylvania; Children's Hospital of Philadelphia, Philadelphia, Pennsylvania; Children's Hospital of Philadelphia, Philadelphia, Pennsylvania; Children's Hospital of Philadelphia, Philadelphia, Pennsylvania

## Abstract

**Background:**

Major Histocompatibility Complex (MHC) and Human Leukocyte Antigen (HLA) loci have strong genetic linkage with type 1 diabetes (T1D) in mice and humans, respectively. The diabetes-prone non-obese diabetic (NOD) strain of mice have a unique MHC-II locus with a distinct MHC-II A molecule (Ag7) and lack expression of the MHC-II E molecule. Expression of this MHC-II A molecule is associated with development of T1D, whereas transgenic restoration of the MHC-II E molecule dominantly protects against T1D. The Silverman laboratory recently demonstrated that MHC-II E molecule expression selects for a diabetes-protective intestinal microbiota in early life though a key knowledge gap remains: how do these two factors — MHC-II molecules and commensal microbiota — work together during a critical early-life period of microbiome and immune system ontogeny to prevent T1D? To address this question, the Silverman laboratory developed a gnotobiotic mouse model by designing a microbial community consisting of 9 intestinal microbes cultured from these diabetes-protected (Ea16/NOD) mice – called “PedsCom”. This gnotobiotic model allows for mechanistic, well-controlled studies of interactions between commensal microbes and the developing immune system.

**Figure:**
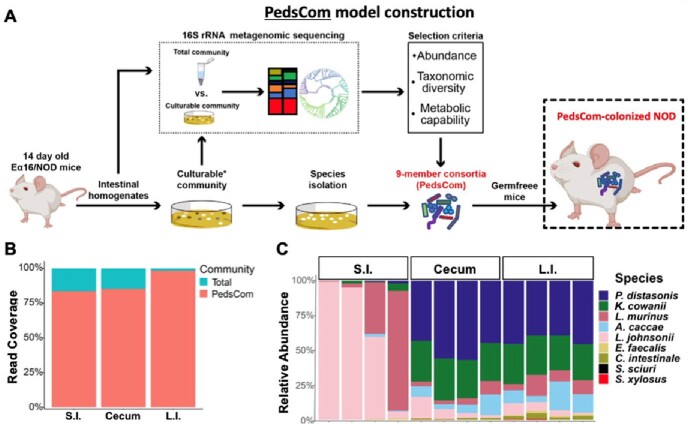

**Figure:**
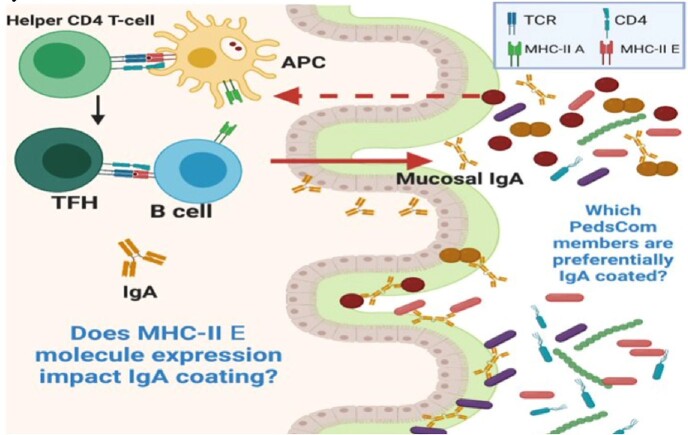

**Methods:**

We used flow cytometry to sort commensal bacteria from PedsCom-colonized NOD and Ea16/NOD mice into IgA-coated and IgA-uncoated populations. We employed species-specific multiplex qPCR to quantify relative abundance of each PedsCom microbe in these sorted populations.

**Figure:**
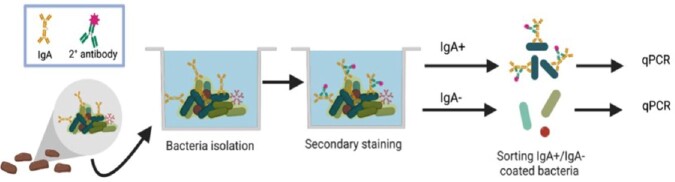

**Results:**

Two microbes, *K. cowanii* and *L. murinus*, were preferentially IgA bound in both NOD and Ea16/NOD mice. *L. johnsonii*, *A. caccae*, and *S. xylosus* were preferentially IgA bound only in the presence of MHC-II E molecule expression. Many of the highly IgA coated microbes (*K. cowanii*, *L. murinus, L, johnsonii*, *and A. caccae*) translocate to the mesenteric lymph nodes.

**Figure:**
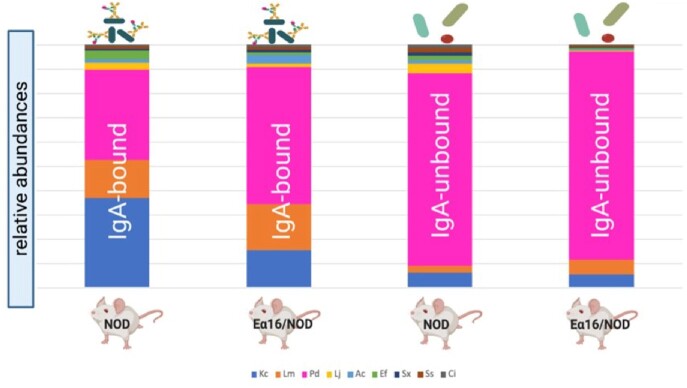

**Figure:**
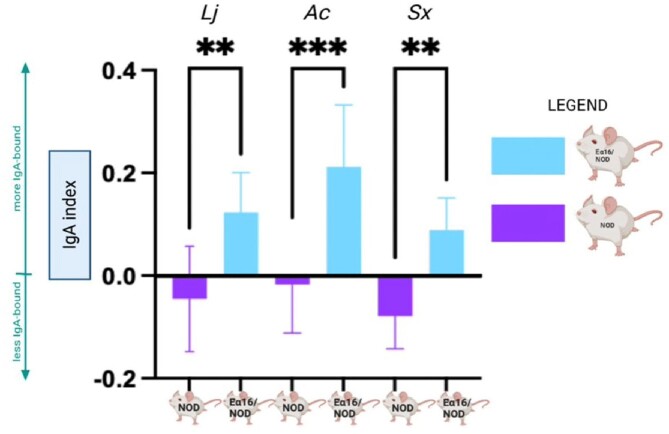

**Conclusion:** We propose that MHC-II expression facilitates specific mucosal IgA responses, that MHC-II E expression allows for additional epitope recognition amongst PedsCom members which may potentiate this effect, and that preferential IgA coating may promote contact with mucosa-associated lymphoid tissues as early steps in tolerogenic immune system ontogeny that protects against development of T1D.

**Disclosures:**

**All Authors**: No reported disclosures

